# Retraction: Quantitative analysis and visualization of literature on acupuncture and related TCM therapies for the treatment of colorectal cancer based on CiteSpace

**DOI:** 10.3389/fonc.2025.1570345

**Published:** 2025-02-14

**Authors:** Frontiers Editorial Office

Following publication, concerns were raised regarding the scientific validity of the article. An investigation was conducted in accordance with Frontiers’ policies.

It was found that the complaints were valid and that the article does not meet the standards of editorial and scientific soundness for Frontiers in Oncology; therefore, the article has been retracted.

This retraction was approved by the Chief Editors of Frontiers in Oncology and the Chief Executive Editor of Frontiers. The authors did not agree to this retraction.

